# COVID-19-Induced Cytokine Release Syndrome Associated with Pulmonary Vein Thromboses, Atrial Cardiomyopathy, and Arterial Intima Inflammation

**DOI:** 10.1055/s-0040-1716717

**Published:** 2020-09-26

**Authors:** Andreas Goette, Markus Patscheke, Frank Henschke, Matthias Hammwöhner

**Affiliations:** 1 Department of Cardiology and Intensive Care Medicine, St. Vincenz Hospital, Paderborn, Germany; 2 Working Group: Molecular Electrophysiology, University Hospital Magdeburg, Magdeburg, Germany; 3 Center for Pathology, Paderborn, Germany

**Keywords:** atria, COVID19, fibrillation, pathology, stroke

## Abstract

Coronavirus disease 2019 (COVID-19) is a viral disease induced by severe acute respiratory syndrome–coronavirus-2 (SARS-CoV-2), which may cause an acute respiratory distress syndrome (ARDS). First reports have shown that elevated levels of inflammatory cytokines might be involved in the development of organ dysfunction in COVID-19. Here, we can present a case of cytokine release syndrome induced by SARS–CoV-2 causing multiorgan failure and death. Of note, we can report on pulmonary vein thromboses as potential source of cerebrovascular embolic events. Furthermore, we present a specific form of an isolated inflammatory atrial cardiomyopathy encompassing atrial myocardium, perivascular matrix, as well as atrial autonomic nerve ganglia, causing atrial fibrillation, sinus node arrest, as well as atrial clot formation in the right atrial appendage. An associated acute glomerulonephritis caused acute kidney failure. Furthermore, all the described pathologies of organs and vessels were associated with increased local expression of interleukin-6 and monocyte chemoattractant protein-1 (MCP-1). This report provides new evidence about fatal pathologies and summarizes the current knowledge about organ manifestations observed in COVID-19.

## Introduction

Cytokine release syndrome (CRS) is a well-described pathological state which may occur after therapy with genetically modified T-cells. 1 In general, CRS is characterized by elevation of several biomarkers such as interleukin (IL)-6, IL-1, and tumor necrosis factor (TNF)-α. Furthermore, ferritin, D-dimer, and C-reactive protein (CRP) are elevated. Clinically, CRS causes fever, nausea, tachypnea, and mental status changes. In more severe forms, CRS is associated with mechanical ventilation, hypotension requiring vasopressor therapy, organ dysfunction, and shock. In addition to cancer therapy, diseases like viral infections have been described to trigger a release of cytokines. 2 3 4 The novel coronavirus Disease 2019 (COVID-19) is a viral disease induced by severe acute respiratory syndrome-coronavirus-2 (SARS-CoV-2) that may cause an acute respiratory distress syndrome (ARDS). At present, the role of CRS in COVID-19 and COVID-19-induced ARDS is not fully understood. 1 5 6 In the present case, we can correlate the clinical course of a COVID-19 patient with systemic biomarkers and histopathological results.

## Case Presentation

**Fig. 1:**
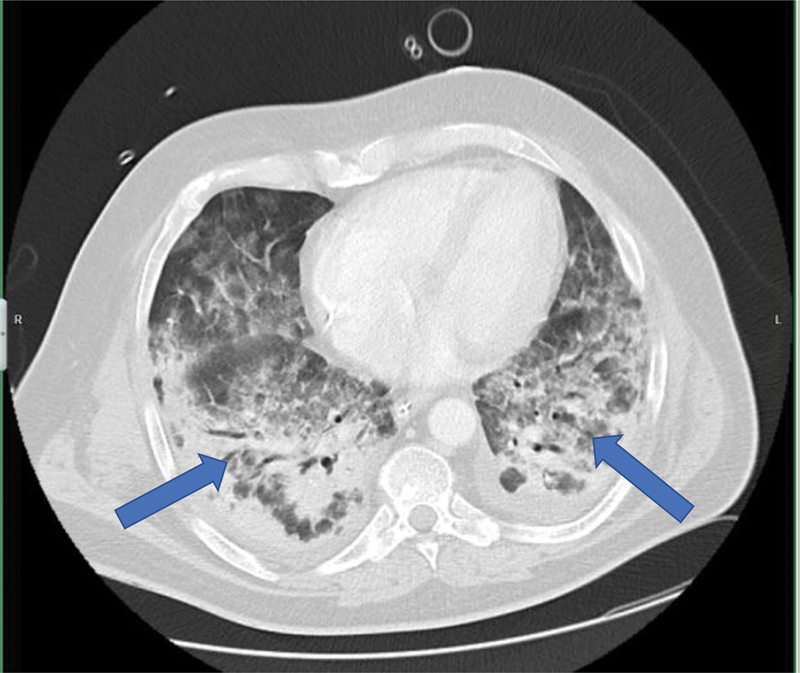
Extensive bilateral lung infiltrates (blue arrows) associated with SARS-CoV-2 infection typical for corona-associated adult respiratory distress syndrome. SARS-CoV-2, severe acute respiratory syndrome–coronavirus-2.

**Fig. 2:**
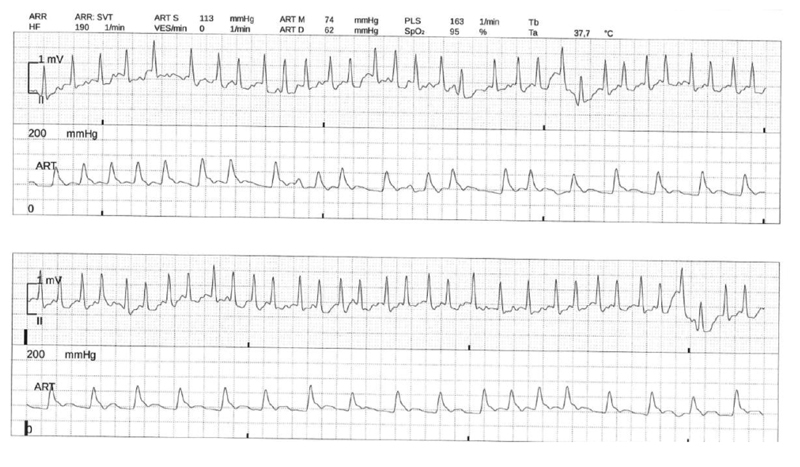
Atrial fibrillation with tachycardic conduction to the ventricles (electrocardiograms both upper rows) as a possible sign for atrial cardiomyopathy. Peripheral pulse deficit as depicted in the arterial pressure measurement due to an increased heart rate of around 190 beats per minute (bpm) with a peripheral pulse deficit (163 bpm; both lower rows).

**Fig. 3:**
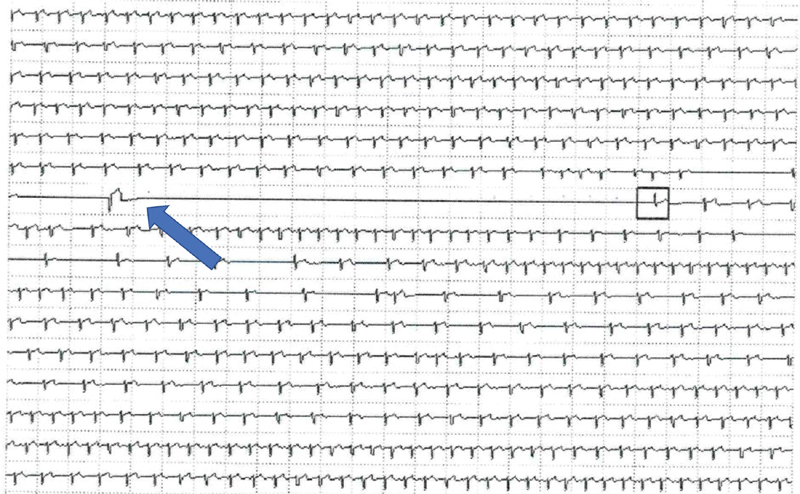
Intermittent sinus node arrest with asystole of >15 seconds (blue arrow).

**Fig. 4:**
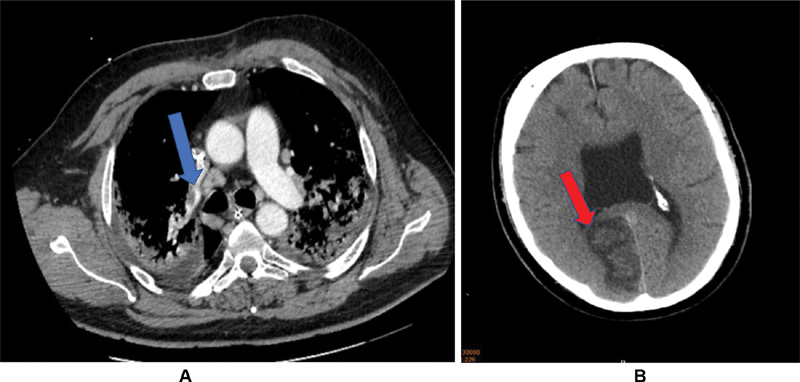
( **A** ) Pulmonary CT scan depicting one of several pulmonal arterial thrombi (right pulmonary subsegment artery, blue arrow). ( **B** ) Cerebral CT scan with evidence of a subacute right posterior territorial ischemic lesion (red arrow). CT, computer tomography.

**Fig. 5:**
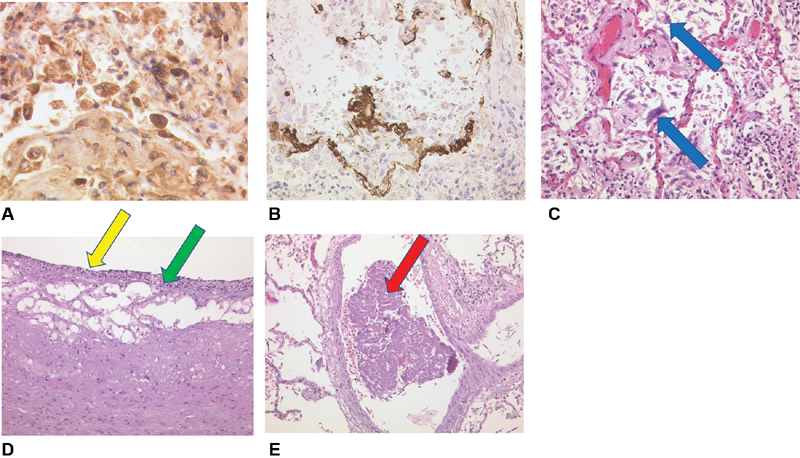
( **A** ) Lung tissue with IL-6 expression (brown colored stain) of plasma cells and alveolar macrophages (pneumocytes II, IL-6, Zytomed), ×80 magnification. ( **B** ) Lung tissue with MCP-1 expression (brown colored stain) in alveolar macrophages (pneumocytes II) and intra-alveolar fibrin (MCP-1, Santa Cruz), ×40 magnification. ( **C** ) The novel coronavirus disease (COVID-19) lung with activated intra-alveolar pneumocytes type II, some with virally altered multiple nuclei (blue arrow), H&E, ×20 magnification. ( **D** ) Left arteria carotis communis with endothelial damage (yellow arrow), necroses, fibrinous exsudation and inflammatory infiltrates of the intimal layer (green arrow), H&E, ×40 magnification. ( **E** ) COVID-19 lung. Early fibrin rich thrombus in a right sided pulmonary vein (red arrow) as a possible atypical origin of thromboembolic stroke, H&E, ×20 magnification. H&E, hematoxylin and eosin; IL, interleukin; MCP, monocyte chemoattractant protein.

**Fig. 6:**
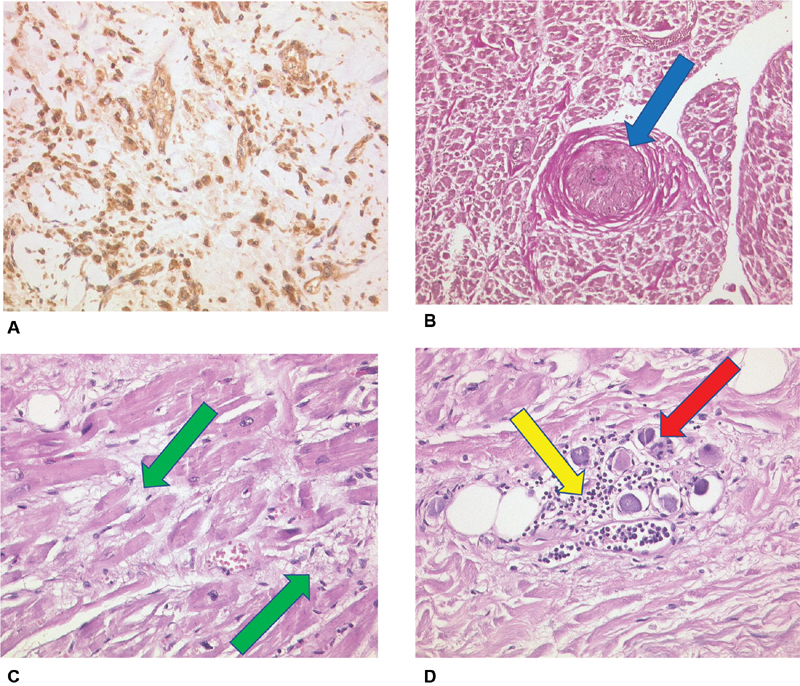
( **A** ) Thrombus in organization with IL-6-expression (brown colored stain) of endothelial cells, Fibroblasts and macrophages (IL-6, Zytomed, ×40 magnification). ( **B** ) Right atrium, stenosing microangiopathy “small vessel disease” (blue arrow), Elastika-van-Gieson (EvG), ×20 magnification. ( **C** ) Right atrium, fresh myocardial necroses (green arrows), H&E, ×40 magnification. ( **D** ) Right atrium, nerval ganglion cells with lymphocyte infiltration (yellow arrow) and viral alterations (red arrow), H&E, ×40 magnification. H&E, hematoxylin and eosin; IL, interleukin.

**Fig. 7:**
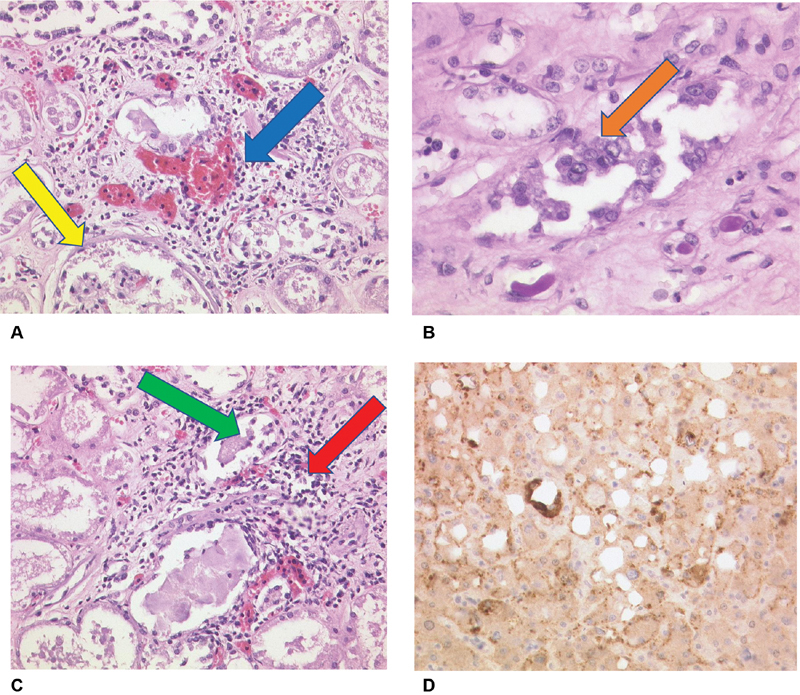
( **A** ) Kidney, viral glomerulonephritis (blue arrow) with cytopathogenic effect of podocytes (yellow arrow), H&E, ×40 magnification. ( **B** ) Kidney, cytopathogenic effect of the tubular epithelial cells, periodic acid–Schiff reaction (PAS), ×80 magnification. ( **C** ) Kidney, necroses of the tubular epithelium (green arrow), interstitial nephritis (red arrow), H&E, ×40 magnification. ( **D** ) Liver with MCP-1 expression in Kuppfer's cells (dark brown colored stain), necroses of hepatocytes (MCP-1, Santa Cruz), ×40 magnification. H&E, hematoxylin and eosin; MCP, monocyte chemoattractant protein.

We report on a 53-year-old male patient with a positive polymerase chain reaction (PCR) nasal swap for SARS-CoV-2, who was hospitalized due to high-grade fever and bilateral lung infiltrates ( Fig. 1 ). Due to rapid deterioration of respiration and development of ARDS, mechanical ventilation of the patient was initiated. On admission to intensive care unit (ICU), the patients also developed atrial fibrillation (AF), which had never been recorded before in this patient ( Fig. 2 ). Due to rapid ventricular rates during AF, the patient was electrically cardioverted and placed on amiodarone intravenous (IV). Anticoagulation was initiated with unfractionated heparin IV with partial thromboplastin time at approximately 50 seconds. Venovenous hemofiltration was initiated because of acute kidney failure with anuria. Even after prone positioning and relaxation, gas exchange deteriorated. Lowest pH was 6.93 with a CO _2_ of 112. Of note, IL-6 reached a maximum level of 2,039 pg/mL (normal value: 0–7 pg/mL), D-dimer was >35 mg/d: (0–5 mg/dL), fibrinogen maxed at 817 mg/dL (170–420 mg/dL), CRP at 38.66 mg/dL (0–0.5 mg/dL), ferritin 3,920 ng/mL (30–400 ng/mL), procalcitonin at 5.47 ng/mL (0–0.5 ng/mL), lactate dehydrogenase reached 1,190 U/L (135–225 U/L), von Willebrand factor (vWF) (FVIII:C)-Activity was 306% (coagulation), vWF-Activity was 447% (turbidimetry), and vWF Antigen was 447% (turbidimetry). Angiotensin II levels were >150 ng/mL (20–40 ng/mL), angiotensin converting enzyme (ACE) decreased to 11 U/L (20–70 U/L), ADAMTS13 (a disintegrin and metalloproteinase with a thrombospondin type 1 motif, member 13) protease activity was reduced to 47%. The patient's blood group was A Rhesus factor positive. Antipospholipid antibodies could not be detected, homocysteine levels were normal with 5.8 µmol/L, and serial heparin-induced thrombocytopenia (HIT) screening tests were negative. At this point, CRS was diagnosed. 5 6 After initiation of 70 mg/day prednisolone, the patients gradually improved. At day 7, a puncture tracheotomy was performed. The procedure was uneventful. But 48 hours later, the patients developed a sinus node arrest with asystole of >15 seconds ( Fig. 3 ). Left ventricular function was not compromised with a normal ejection fraction on echocardiography. A temporary pacemaker wire was inserted through the left jugular vein after two more episodes of sinus node arrest for >10 seconds. On day 11, a computed tomography (CT) scan of the brain and chest revealed that the patient had suffered multiple pulmonary embolisms ( Fig. 4A ) and multiple thromboembolic strokes of which the largest was in the right posterior hemisphere ( Fig. 4B ). Due to repetitive episodes of sinus node arrest, a permanent pacemaker was consecutively implanted. Twenty-four hours after pacemaker implantation, the patient developed a fatal hemorrhagic shock due to a massive pulmonary bleeding. Autopsy confirmed a severe form of SARS–CoV-2 induced ARDS ( Fig. 5A ). Of note, total lung weight was 3 kg. Immunohistochemistry revealed local overexpression of IL-6 ( Fig. 5B ) and MCP-1 ( Fig. 5C ) in pulmonary macrophages and alveolar epithelial cells type II. Furthermore, arterial endothelial damage, necrosis, fibrinous exsudation, and inflammatory infiltrates of the intimal layer were present, that is, in the left carotid artery ( Fig. 5C ). Atypical locations of thrombus formation included a pulmonary vein of the right lung ( Fig. 5D ) and the right atrial appendage. Histologically, not only subendothelial vascular walls but also atrial walls were invaded by inflammatory cells. Interestingly, IL-6 overexpression could be found within vascular thrombi, adherent endothelial cells, and fibroblasts ( Fig. 6A ). In the right atrium signs of inflammatory microangiopathy (“small vessel disease,” Fig. 6B ) in conjunction with mild lymphocytic myocarditis and early myocardial necroses were present ( Fig. 6C ). Interestingly, as a possible cause for AF and sinus node dysfunction, histological examination also revealed that ganglionated right atrial plexi were infiltrated by lymphocytes and virus-infected ganglial cells could be observed ( Fig. 6D ). In the kidneys a glomerulonephritis with cytopathogenic effect of the podocytes, tubular epithelial necroses with cytopathogenic effect of tubular cells ( Fig. 7A, B ) and interstitial nephritis could also be detected ( Fig. 7C ). Furthermore, involvement of the liver was also shown by overexpression of MCP-1 in Kupffer's cells ( Fig. 7D ).

In summary, the present case shows that severe COVID-19 induces CRS associated with ARDS, acute kidney failure, liver pathologies, vascular intimal inflammation, pulmonary arterial, and venous thromboses and an inflammatory atrial cardiomyopathy. In particular, the presence of unusual clot formation in the right atrial appendage, but also loosely detached clots within the pulmonary venous system are novel findings, since the latter might be a source of systemic stroke in COVID-19 patients. Of note, the venous clots could not be detected by conventional contrast CT scans, which revealed the presence of embolisms in the pulmonary artery in the presented patient.

## ARDS and COVID-19

In COVID-19 patients with ARDS, there are inflammatory infiltrates of alveolar and interstitial tissue, increased vascular permeability, as well as microcirculatory flow abnormalities, due to thrombus formation within the capillaries. 7 ARDS appears to occur in 5 to 30% of COVID-19 patients. 8 A recent, to date only on preprint servers and not yet peer reviewed large scale study with a genome-wide association analysis including 1,980 patients and 8,582,968 single-nucleotide polymorphisms identified blood group A Rhesus factor positive, the blood group of our patient, as a risk marker for respiratory failure in COVID-19 patients. 9 Ellinghaus et al also found a protective effect for blood group O. 9

Increased activation of the clotting system appears as one hallmark of COVID-19. 10 There is growing evidence for an impact of the activated coagulation factor X (FXa) in inflammatory lung diseases. 11 A study investigated the effects of FXa on epithelial lung cells (A549 cell line). 12 Of note, FXa increases expression of cytokines in alveolar epithelial cells, which can be prevented by an inhibitor of protease-activated receptor 1, vorapaxar. Interestingly, the presented patient developed arterial and venous pulmonary microthrombi in the circulatory tree and pulmonary bleeding due to increased capillary permeability. These venous and arterial thrombotic vascular occlusions in the lungs were found despite the constant use of effective heparin IV in the current case. Further studies are warranted to assess the effect of FXa inhibitors to prevent clot formation in the lungs of COVID-19 patients.

## Activation of Clotting System in COVID-19

Viral infections, such as SARS-CoV-2, may induce systemic inflammatory pathways. 13 Activation of host immune systems can cause activation of the plasmatic clotting system resulting in thrombogenesis called thromboinflammation or immunothrombosis. 14 15 Importantly thrombotic complications have been described in 5 to 23% of COVID-19 cases. 16 The clotting system can be activated by multiple procoagulant pathways. Activated platelets, mast cells, and tissue factor or FXII may induce the intrinsic coagulation pathway. 17 Cytokines may cause endothelial injury with endothelial and intimal necrosis and expression of adhesion molecules, which might later on be associated with thrombocytopenia. 18 19 Subsequent decline in clotting factors occurs with enhanced fibrinolysis during severe infections, which is also characterized by elevated D-dimers. 20 Activation of the clotting system with decline of clotting factors and thrombocytopenia has been described to occur in patients in later stages of COVID-19. 20 21

Massive systemic inflammation has been described in patients with SARS-CoV-2 infections. This cytokine release syndrome (CRS) is characterized by elevated levels of IL-6, increased CRP, ferritin, and elevated fibrinogen. 22 A report of COVID-19 patients in China found elevated plasma concentrations of inflammatory markers in particular in patients with severe infections. 23 In COVID-19, elevated D-dimer levels have been associated with thromboembolism and worse prognosis. 24 25 26 Tang et al showed elevated fibrinogen levels. 20 Another study found elevation of fibrinogen, D dimer, and IL-6 levels in patients with COVID-19-induced ARDS. 11 In COVID-19 patients, 71.4% of nonsurvivors and 0.6% survivors met the criteria of disseminated intravascular coagulation. 20

Initial results have described antiphospholipid antibodies as a cause of coagulopathy in some patients. 27 Unlike other RNA viruses inducing hemorrhagic manifestations (hemorrhagic fever viruses), SARS-CoV-2 has not been reported to result in significant bleeding. 28 However, the present case showed fatal diffuse pulmonary bleeding, which is one of the first descriptions that COVID-19 might cause substantial hemorrhagic manifestations besides thrombogenesis in arteries, veins and as in our case in the heart.

## Vascular Intimal Dysfunction in COVID-19

Recent reports have shown the occurrence of an “endotheliopathy,” 29 which contributes to microcirculatory changes in SARS-CoV-2 infections. 30 The receptor for viral adhesion is the ACE-2 receptor, causing inflammatory cell infiltration, endothelial cell apoptosis, and microvascular prothrombotic effects. 29 The ACE2 receptors are expressed in different organs like lung, heart, kidneys, and endothelial cells. Thus, SARS-CoV-2 can effect endothelial cells in many different organs via ACE2 binding. This finding is supported by the present case because we found a generalized inflammation of the vascular endothelium but also the vascular intima causing endothelial shedding, thrombus formation and diffuse bleeding, particularly in the lungs. Recent reports suggest that viral inclusions within endothelial cells and sequestered mononuclear and polymorphonuclear cellular infiltration might induce endothelial apoptosis. 29 Nevertheless, CRS has been clearly described to cause massive endothelial dysfunction and microcirculatory flow abnormalities associated with multiple clots within the capillaries. As a result, microcirculatory dysfunction in solid organ may occur causing organ failure in patients with COVID-19. In the present case, we can clearly show that the intima of the carotid arteries is substantially invaded by inflammatory cells causing necrosis of the intima and endothelial denudation. Of note, we can show overexpression of cytokines in areas of vascular necrosis and thrombosis. Thus, the vascular alterations are much more complex than isolated endothellopathy. Furthermore, we can show for the very first time that the atrial tissue of the right atrium is affected by the systemic inflammatory process as well. In the present case, we can show that COVID-19 can induce the occurrences of ARDS, which was associated with pulmonary embolism, as well as thrombogenesis, in pulmonary veins and the right atrial appendage. Thus, further studies are warranted to assess the use of antiplatelet therapy and/or oral anticoagulant therapy in patients with this condition to prevent organ ischemia.

## Inflammatory Atrial Cardiomyopathy

The term “atrial cardiomyopathy” has been introduced by a worldwide consensus document in the year 2016. 31 Inflammatory changes of atrial tissue have been described in the presence of various form of myocarditis or toxic agents. Here, we can describe for the first time that COVID-19 induces an inflammatory atrial cardiomyopathy that caused sinus node dysfunction and AF. In the present case, it remains unclear if AF was causally related to the occurrence of stroke, since severe changes at the endothelium could be documented in the carotid artery and thromboses were also found in the pulmonary veins and the right atrial appendage. In contrast to the significant atrial alterations, histological exam of left ventricular tissue showed only mild subendothelial scarring without significant lymphocytic infiltration. Accordingly, left ventricular function was normal throughout the course of hospitalization monitored by echocardiography. Studies have recently described mild lymphocytic myocarditis and signs of epicarditis in the ventricles of COVID-19 patients. 32 To our knowledge, however, the manifestation of COVID-19 in cardiac ganglionated plexi with clinical manifestation of sick sinus syndrome has not been described before. It remains unclear at this point if parts of right atrial clots may have contributed to embolic events in the pulmonary arteries.

## Mechanisms of Stroke in COVID-19

Several reports have shown an increased rate of pulmonary thromboembolic events, stroke, and systemic embolism in COVID-19. 33 Neurologic manifestations might occur in up to 36%. 33 These events appear to be related to activation of the plasmatic clotting system, platelet activation, and vascular intimal dysfunction or endothelial denudation causing local thrombus formation and organ ischemia. The present case further suggests that AF and the development of pulmonary vein thrombosis might also be additional factors that may contribute to the development of stroke in COVID-19 patients. There are rare reports on patients with pulmonary arteriovenous shunts, which might become clinically apparent by repetitive cerebral strokes. 34 Thus, the pulmonary venous system might be the source of thrombus formation with cerebral clot embolization. The present case also shows that COVID-19-induced ARDS is associated with massive clotting in the pulmonary microcirculation. In addition, clot formation may also occur in pulmonary veins, and therefore, pulmonary venous clots might be a source for systemic embolism and stroke in COVID-19.

## Novel Therapeutic Approaches in COVID-19

**Fig. 8:**
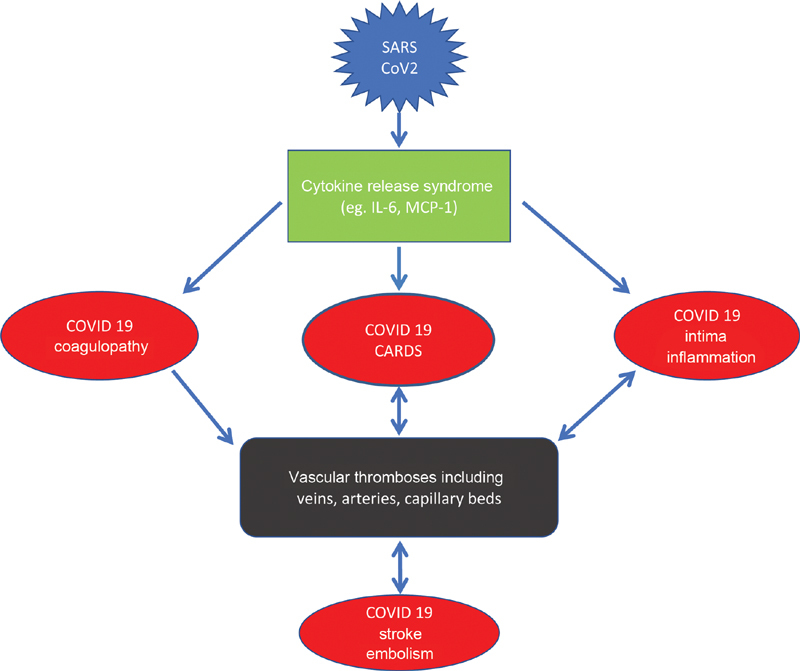
Summary figure about cytokine release syndrome (CRS) in COVID-19. CRS is associated adult respiratory stress syndrome (ARDS) of the lungs, vascular intimal inflammation and coagulopathy with increased incidence of thromboembolic complications. COVID-19, novel coronavirus disease 2019; CRS, cytokine release syndrome; MCP, monocyte chemoattractant protein; SARS-CoV-2, severe acute respiratory syndrome–coronavirus-2.

In the lack of an effective vaccine for preventing severe medical conditions associated with SARS-CoV-2 infections ( Fig. 8 ), several novel therapeutic approaches have been proposed, encompassing antivirals, antimalarials, and immunomodulators that have shown activity against SARS-CoV-2. 35 36 In particular, hydroxychloroquine, remdesivir, interferon β-1b, lopinavir-ritonavir, ribavirin, favipiravir, arbidol, tocilizumab, and bevacizumab have been investigated. 35 Mostly, however, these therapies have been evaluated in single cases or small-scale studies. Remdesivir (Gilead Science), a nucleotide analogue prodrug that inhibits viral RNA polymerases, was originally evaluated for treatment in Ebola Virus disease, but it has also shown in vitro activity against SARS-CoV-2. Preliminary data show that remdesivir may be beneficial in the early phase of SARS-CoV-2 infection. 37 Hung et al have recently evaluated a combined antiviral and imunomodulator therapy with interferon β-1b, lopinavir-ritonavir, and ribavirin in a multicenter, open label, randomized phase-IIb trial in COVID-19 patients. 38 The authors conclude that early treatment with the triple combination therapy may successfully reduce viral shedding and hence hospitalization duration. Zhagn et al reported on successfully treating a severely ill COVID-19 patient with dose adjustment methylprednisolone according to inflammation parameters and T-cell count. 39 In our case, however, despite early initiation of corticosteroid therapy, the patient developed a severe form of CRS with highly elevated IL-6 levels. Early reports show that tocilizumab, an IL-6 inhibitor, may be beneficial in this patient population. 40 41 42 Furthermore, due to the observed alterations at the arterial, venous, and atrial endothelium further studies are warranted to assess the optimal anticoagulative strategy including different anticoagulants and potential combinations of anticoagulants with antiplatelet drugs.

## Conclusion

COVID-19 is associated with development of CRS, which contributes to fatal damage of solid organs. Massively increased IL-6 levels and MCP-1 appear a systemic blood marker of CRS. In addition to COVID-19-induced ARDS, CRS might be associated with pulmonary artery, as well as vein thromboses, atrial fibrillation, sinus node dysfunction, right atrial clot formation, and inflammatory invasion of autonomic atrial nerve ganglia. Furthermore, hepatitis and glomerulonephritis might occur at a very early stage of the disease leading to acute organ failure within days of COVID-19 ( Fig. 8 ). Studies are warranted to examine, if therapeutic agents against CRS-like IL-6 receptor antagonist tocilizumab and/or anticoagulants plus antiplatelet therapy are useful to treat patients with severe COVID-19.
